# Traces of pandemic fluoroquinolone-resistant *Escherichia coli* clone ST131 transmitted from human society to aquatic environments and wildlife in Japan

**DOI:** 10.1016/j.onehlt.2024.100715

**Published:** 2024-03-23

**Authors:** Toyotaka Sato, Kojiro Uemura, Mitsuru Yasuda, Aiko Maeda, Toshifumi Minamoto, Kazuki Harada, Michiyo Sugiyama, Shiori Ikushima, Shin-ichi Yokota, Motohiro Horiuchi, Satoshi Takahashi, Testuo Asai

**Affiliations:** a Laboratory of Veterinary Hygiene, Faculty of Veterinary Medicine, Hokkaido University, Sapporo, Japan; b Graduate School of Infectious Diseases, Hokkaido University, Sapporo, Japan; c One Health Research Center, Hokkaido University, Sapporo, Japan; d Department of Microbiology, Sapporo Medical University School of Medicine, Sapporo, Japan; e Department of Infection Control and Laboratory Medicine, Sapporo Medical University School of Medicine, Sapporo, Japan; f Department of Human Environmental Science, Graduate School of Human Development and Environment, Kobe University, Kobe, Japan; g Joint Department of Veterinary Medicine, Tottori University, Tottori, Japan; h The United Graduate School of Veterinary Sciences, Gifu University, Gifu, Japan; i Division of Laboratory Medicine, Sapporo Medical University Hospital, Sapporo, Japan

**Keywords:** Antimicrobial resistance, *Escherichia coli*, Fluoroquinolone resistance, ST131

## Abstract

Transmission of antimicrobial-resistant bacteria among humans, animals, and the environment is a growing concern worldwide. The distribution of an international high-risk fluoroquinolone-resistant *Escherichia coli* clone, ST131, has been documented in clinical settings. However, the transmission of ST131 from humans to surrounding environments remains poorly elucidated. To comprehend the current situation and identify the source of ST131 in nature, we analyzed the genetic features of ST131 isolates from the aquatic environment (lake/river water) and wildlife (fox, raccoon, raccoon dog, and deer) and compared them with the features of isolates from humans in Japan using accessory and core genome single nucleotide polymorphism (SNP) analyses. We identified ST131 isolates belonging to the same phylotype and genome clusters (four of eight clusters were concomitant) with low SNP distance between the human isolates and those from the aquatic environment and wildlife. These findings warn of ST131 transmission between humans and the surrounding environment in Japan.

## Highlights

• Fluoroquinolone-resistant *E. coli* pandemic clone, ST131, is isolated from aquatic environments and wildlife.
• Genetic similarity of ST131 between humans and aquatic environments and wildlife was observed.
• It suggests and warns the ST131 overflow from human society to aquatic environments and wildlife.

## 1 Text

Extraintestinal pathogenic *Escherichia coli* (ExPEC) is the most common pathogenic gram-negative bacteria that causes urinary tract and bloodstream infections in clinical settings. The international high-risk fluoroquinolone-resistant ExPEC clone, sequence type (ST) 131, has caused clinical concerns in the past two decades owing to its pathogenicity, pandemic spread, and frequent co-resistance to third-generation cephalosporins by producing CTX-M-type extended-spectrum β-lactamases (ESBL) [1]. Certain ST131 strains reportedly exhibit multidrug resistance including carbapenem resistance and/or resistance to last-line drugs, including colistin and tigecycline [[1], [2], [3]]. In recent years, ST131 prevalence has also increased in companion animals (dogs and cats) [4,5]; however, it remains lower than that in humans [6]. ST131 was isolated among ESBL-producing *E. coli* from wildlife in Japan [[7], [8], [9]]. The spread of ST131 among wildlife and subsequently to the environment is a cause of concern [10] because such dissemination makes the eradication of ST131 more challenging by establishing its circulation throughout human society and the surrounding environment. In the current study, we analyzed ST131 isolates from the natural environment (aquatic environment and wildlife) and humans in Japan to estimate the current situation and future possibilities of ST131 dissemination across humans and the surrounding environment.

We identified 16 ST131 isolates from feces of wildlife (5 isolates from raccoon dogs, deer, fox, and raccoon [[7], [8], [9]]) and from aquatic environments (11 isolates from river and lake water) between 2016 and 2021 using the isolation method described previously [11] (Table 1). The regions from where the isolates were derived are in neighboring prefectures in Japan, except the Kanagawa prefecture. The ST131 and the clades were identified using multiplex PCR, as described previously [12]. The antimicrobial susceptibility was tested using the broth microdilution method, and a minimum inhibitory concentration > 1 mg/L for ciprofloxacin was defined to indicate fluoroquinolone resistance according to the Clinical and Laboratory Standards Institute [13].

**Table 1:** Origin and characterization of environmental ST131 isolates used in the current study.

Origin	Material	Host	Strain ID	Year	Location	CIP MIC (mg/L)	Serotype	MLST	Pylotype	Cluster	Reference
Wild animals	Feces	Raccoon dog	H43	2016	Kanagawa	>4	O25:H4	131 (C1-nM27)	I	e	[7]
	Feces	Raccoon dog	H81	2017	Kanagawa	1	O16 H5	131	II	ND	
	Feces	Deer	GifuUI69	2018	Nara	>4	O25:H4	131 (C2)	I	h	This study
	Feces	Fox	wlxctx4	2021	Gifu	>4	O25:H4	131 (C1-nM27)	I	e	[9]
	Feces	Raccoon dog	WLCTX8	2021	Gifu	>4	O25:H4	131 (C1-M27)	I	f	[8]
Environmental water	Water	Ijira river	GifuUK25a	2018	Gifu	>4	O25:H4	131 (C1-nM27)	I	e	This study
	Water	Ijira river	GifuUK57	2018	Gifu	>4	O25:H4	131 (C1-M27)	I	c	
	Water	Ijira river	GifuUKF44a	2019	Gifu	>4	O25:H4	131 (C1-nM27)	I	e	
	Water	Ijira river	KF046	2019	Gifu	0.5	O16:H5	131	II	ND	
	Water	Ijira river	KF128	2019	Gifu	1	O16:H5	131	II	ND	
	Water	Ijira river	KF134	2019	Gifu	1	O16:H5	131	II	ND	
	Water	Ijira river	KF337	2021	Gifu	2	H5	131	II	ND	
	Water	Lake Biwa	GifUBW5	2021	Shiga	>4	O25:H4	131 (C1-nM27)	I	e	
	Water	Lake Biwa	GifUBW25	2021	Shiga	>4	O25:H4	131 (C1-M27)	I	c	
	Water	Lake Biwa	GifuUBW32	2021	Shiga	>4	O25:H4	131 (C1-M27)	I	c	
	Water	Lake Biwa	GifuUBW83	2021	Shiga	>4	O25:H4	131 (C1-M27)	I	c	

CIP, ciprofloxacin: MIC, minimum inhibitory concetrration: MLST, multilocus sequence typing.

To compare the genetic background of environmental isolates, we also analyzed 57 fluoroquinolone-resistant *E. coli* isolates derived from urine samples of patients in Gifu and Shiga prefectures from 2016 to 2021. These isolates were obtained during clinical diagnoses using 5% Sheep Blood Agar (Becton Dickinson and Company, Franklin Lakes, NJ). Identification of *E. coli* was performed using MALDI Biotyer (Becton Dickinson and Company). Whole-genome sequencing of DNA library from fluoroquinolone-resistant *E. coli* isolates prepared using using Illumina DNA prep kit was performed using MiSeq or iSeq (Illumina DNA prep, Illumina, San Diego, CA). Quality check was performed using FastQC, and trimming and *de novo* assembly were performed using a CLC Genomics Workbench (Qiagen, Hilden, Germany). ST131 and serotypes were identified using multilocus sequence typing (MLST 2.0) and SeroTypeFinder 2.0, respectively, at the Center for Genomic Epidemiology (https://www.genomicepidemiology.org). The assembled genomes were annotated using Prokka 1.14.6 (https://github.com/tseemann/prokka) [14], followed by pan- and accessory genome analysis using Roary (https://github.com/sanger-pathogens/Roary) [15], together with 65 complete *E. coli* ST131 genomes with complete epidemiological information (origin, year of isolation, and country) available in the NCBI database. The thresholds for the phylotypes and clusters were defined at tree-scale values of 0.01 and 0.005, respectively. Phylogenetic analysis based on the core genome single nucleotide polymorphisms (SNP) was performed using the kSNP4 program [16], and Kmer size 17 and *E. coli* EC958 (Accession number was HG941718.1) were used as the reference. Pairwise SNP distance matrix was generated using snp-dists 0.8.2 (https://github.com/tseemann/snp-dists) and illustrated as a matrix heatmap using SRplot (https://www.bioinformatics.com.cn/en).

In the current study, except for six ST131 clade C2 isolates, the others were ST131 clade C1, which consists of subclades C1-nM27 and C1-M27, carrying CTX-M-type ESBL genes (*bla*_CTX-M-14_ and *bla*_CTX-M-27_, respectively) [17]. Clade C1 is a distinct ST131 cluster that has spread throughout Japan since the 2000s [17]. Previous studies have reported the isolation of clade C1 from companion animals in Japan [4], as observed in the current study (Fig. S1). The isolation of clade C1 from environment has also been reported in other countries (for example, from water birds, rivers, and lakes in Central Europe and Switzerland) [17]. However, the appearance was low, and genetic relationships with human ST131 remain unclear from the study. In addition, the isolation of ST131 clade B that carries the *fimH22* allele from poultry and humans has been reported, suggesting the zoonotic potential [10,18], which was not isolated in this study.

Using Roary, we observed a total of 1590 core and 14,533 accessory genes. Using the accessory genome analysis, ST131 isolates were divided into two phylotypes (I and II) and eight clusters (from a to h) (Fig. S1). Phylotype I only comprised Japanese isolates (*n* = 51). The ST131 isolates derived from river/lake water and wildlife included 11 and 5 isolates belonging to phylotypes I and II, respectively (Table 1). Among them, 11 isolates of phylotype I were serotype O25:H4 and belonged to four clusters, c (*n* = 4 isolates), e (*n* = 5), and f and h (*n* = 1, respectively) (Table 1 and Fig. S1), as determined using accessory genome analysis. This phylotype from river/lake water and wildlife-derived isolates in Japan consisted of three ST131 clades, C1-M27 (clusters c and f), C1-nM27 (cluster e), and C2 (cluster h) (Table 1). The five isolates of phylotype II were identified as another ST131, serotype O16:H5 (Table 1). Therefore, these observations indicate that ST131 spreads in the aquatic environment and wildlife in Japan at a certain frequency.

Among human isolates derived from urine samples, 41 of 57 fluoroquinolone-resistant *E. coli* isolates (71.9% of the total) were identified as ST131 (O25:H4), suggesting the dominance of ST131 in these prefectures, consistent with that in other regions of Japan [6,16]. Among them, 38 isolates (66.7%) belonging to phylotype I comprised the following seven clusters: a (*n* = 3 isolates), b (*n* = 3), c (*n* = 5), e (*n* = 6), f (*n* = 2), g (*n* = 3), and h (*n* = 16) (Table 2 and Fig. S1). The phylotype I of human ST131 isolates also comprised three ST131 clades, C1-M27 (belonging to accessory genome-based clusters a, b, c, f, g, and h), C1-nM27 (belonging to clusters e and h), and C2 (belonging to cluster h) (Table 2). The remaining three ST131 isolates derived from humans belonged to phylotype II and were identical to clade C2 (Table 2 and Fig. S1).

**Table 2:** Phylotype of ST131 isolates from human patients from Gifu and Shiga prefectures.

Pylotype	Cluster	MLST	2016	2017	2018	2021	Total	CIP MIC (mg/L)
I	a	131 (C1-M27)			3		3	>2
	b	131 (C1-M27)				3	3	>2
	c	131 (C1-M27)	1		2	2	5	>2
	e	131 (C1-nM27)	5	1			6	>2
	f	131 (C1-M27)				2	2	>2
	g	131 (C1-M27)			1	2	3	>2
	h	131 (C1-M27)	1		3		4	>2
	h	131 (C1-nM27)	2		7	3	12	>2
II	ND	131 (C2)			2	1	3	>2
Total			9	1	15	13	41	

MLST, multilocus sequence typing: CIP, ciprofloxacin: MIC, minimum inhibitory concetrration.

Collectively, we revealed the concurrence of ST131 isolates from humans, water environments, and wildlife in several accessory-genome-based clusters (four of eight clusters, c, e, f, and h, in phylotype I). This observation suggests the spread and transmission of ST131 between human society and the surrounding nature in Japan, although a direct epidemiological relationship for each sample could not be established.

Core genome SNP of ST131 phylotype I showed clustering of some isolates from humans, river/lake, and wildlife (Fig. S2A). In particular, some ST131 strains exhibited low SNP distance (SNP number ranging from 18 to 62) between the isolates from humans and river and lake water/companion animals (Fig. S2B). The low SNP distance between ST131 isolates from humans and wild birds/environment (number of SNP number ranging from 16 to 75) was reported in Australia around 2019 [10]. These observations consistently indicated similar genetic backgrounds between some ST131 (mainly clade C1) derived from humans, wildlife, and river/lake water samples and transmitted among each other in some countries.

Accordingly, we suggest that ST131 disseminates from humans to the aquatic environment/wildlife based on the following observations in Japan: i) identical ST131 phylogeny and clade C1 between humans, wildlife, and river/lake water, and previous observations (the current study); ii) higher prevalence of fluoroquinolone-resistant *E. coli* in humans (approximately 40.4% (154,100/381,447) isolates in patients [19] but 9.6% (35/336 isolates) in wildlife [8]) and clade C1 dominance in humans (>50% of total fluoroquinolone-resistant *E. coli*; Fig. S1, [6]); iii) the occurrence of ST131 clade C1 in human isolates around 2004 [17] but no reports from the nature prior to the report from humans in Japan; and iv) a recent report documenting the isolation of ST131 in wastewater in other regions of Japan [20].

In the current study, the lake water-isolated ST131 was obtained from Lake Biwa, the largest lake in Japan. Lake Biwa is the source of approximately 450 rivers, including 117 class A rivers, and thus, the flow is the primary source of tap water for 14.5 million people, covering approximately 11.5% of Japan's population, and agricultural and industrial water in Japan. Therefore, these results imply that ST131 overflowed from human society to the aquatic environment/wildlife *via* the effluent. This dissemination may also apply to another ST131 clade, C2, isolated from humans and deer and belonging to an identical cluster (cluster h in Fig. S1). The limitation of this study is that the observations made were circumstantial and more direct evidence is required to confirm the transmission.

In conclusion, the fluoroquinolone-resistant *E. coli* ST131 isolated from aquatic environments and wildlife was genetically associated with ST131 clone spreading in clinical settings in Japan. This finding is a warning regarding the overflow and pollution of pandemic ST131 from human society to the surrounding environments, warranting continuous surveillance based on the One Health approach.

## Ethical approval statement

This study was approved by the Ethics Committee for Animal Research and Welfare of Gifu University, Japan (approval numbers 17,055 and 17,110), the Ethics Committee for Academic Research of Captured Animals in Gifu Prefecture (approval number 269), and the Gifu University Graduate School of Medicine Medical Research Ethics Review Committee (No. 29–285).

## Declaration of competing interest

The authors declare that they have no known competing financial interests or personal relationships that could have appeared to influence the work reported in this paper.

## Data Availability

The accession numbers of used isolates are described in Fig. S1 and are available in the NCBI database (https://www.ncbi.nlm.nih.gov).

## Appendix A Supplementary data

Supplementary material Supplemental Fig. S1. Accessory genome analysis of ST131 isolated in Japan and other countries. Isolates highlighted in color are fluoroquinolone-resistant *Escherichia coli* isolates derived from specific regions (three neighboring prefectures, Gifu, Shiga, and Nara) of Japan. Isolates are shown in yellow (urine samples from human patients), brown (cat and dog feces), green (wildlife feces), and blue (river and lake water). The thresholds for the phylotypes and clusters were defined at tree-scale values of 0.01 and 0.005, respectively. ND, not determined; ST, sequence type. Parentheses in the ST and country columns indicate ST131 clades and prefectures, respectively. The phylogeny of the pan-genome analysis is not shown because it could not classify ST131 into several phylotypes and/or clusters owing to the high genomic similarity of identical clones. Supplemental Fig. S2. Core genome SNP analysis of ST131 isolated in Japan and other countries ST131 isolates belonging to phylotype I were subjected to accessory genome analysis (Supplemental Fig. S1) to determine the genetic relationship of ST131 isolates with a higher resolution using core genome SNP analysis. The results are shown as a phylogenetic tree based on core genome SNP (A) and matrix heatmap of the pairwise SNP distance (B). Isolates highlighted in color are fluoroquinolone-resistant *Escherichia coli* isolates derived from specific regions (three neighboring prefectures, Gifu, Shiga, and Nara) of Japan. Isolates are shown in yellow (urine samples from human patients), brown (cat and dog feces), green (wildlife feces), and blue (river and lake water). In the pairwise SNP distance, the matrix heatmap shows the number of SNP in the ST131 strains. Supplementary material
