# Supplementary figures and images for: Traces of pandemic fluoroquinolone-resistant Escherichia coli clone ST131 transmitted from human society to aquatic environments and wildlife in Japan
Source: One Health. 2024 Mar 23;18:100715. doi: 10.1016/j.onehlt.2024.100715 (PMC11247291; doi:10.1016/j.onehlt.2024.100715)

## Slide 1
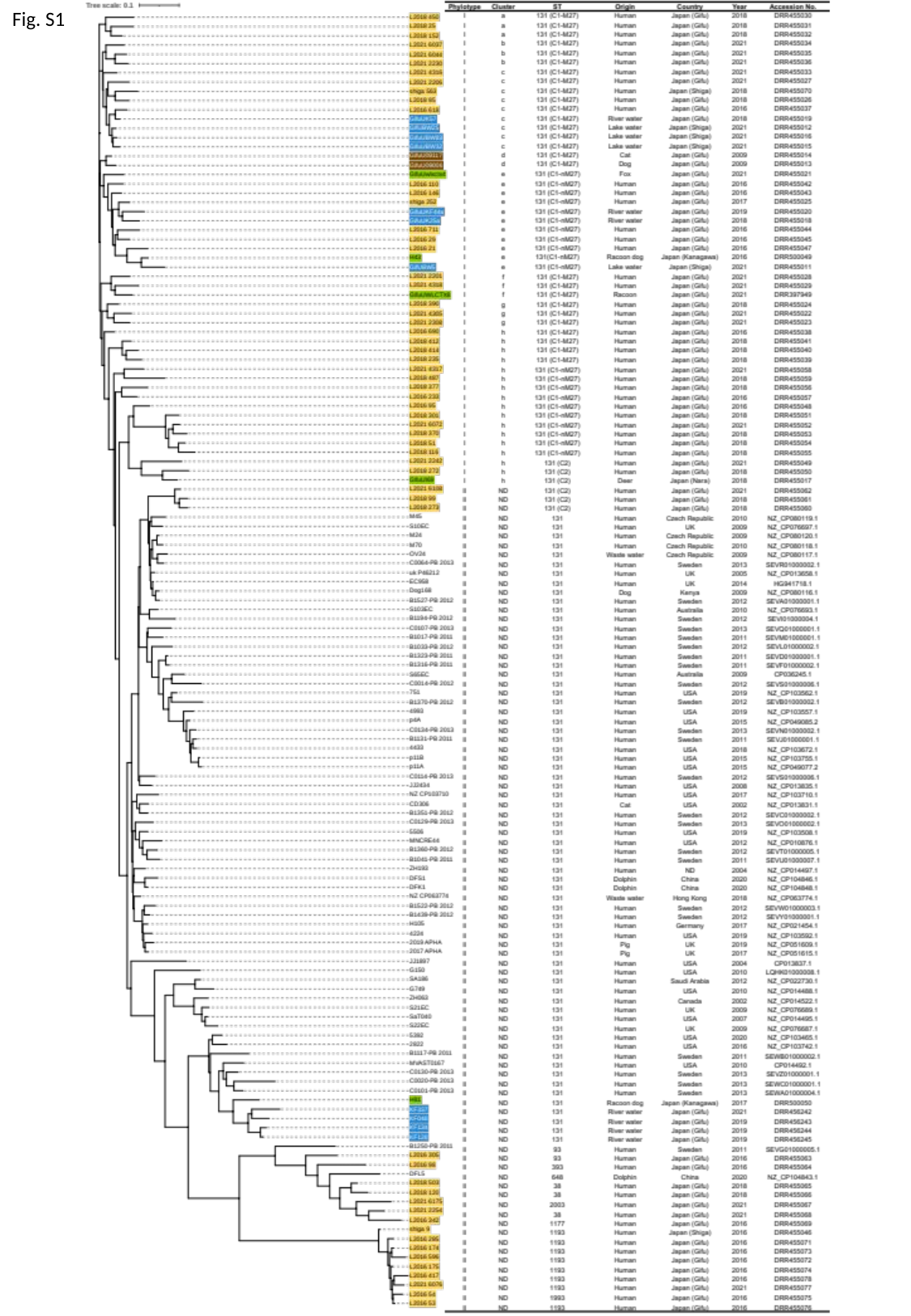

Fig. S1

## Slide 2
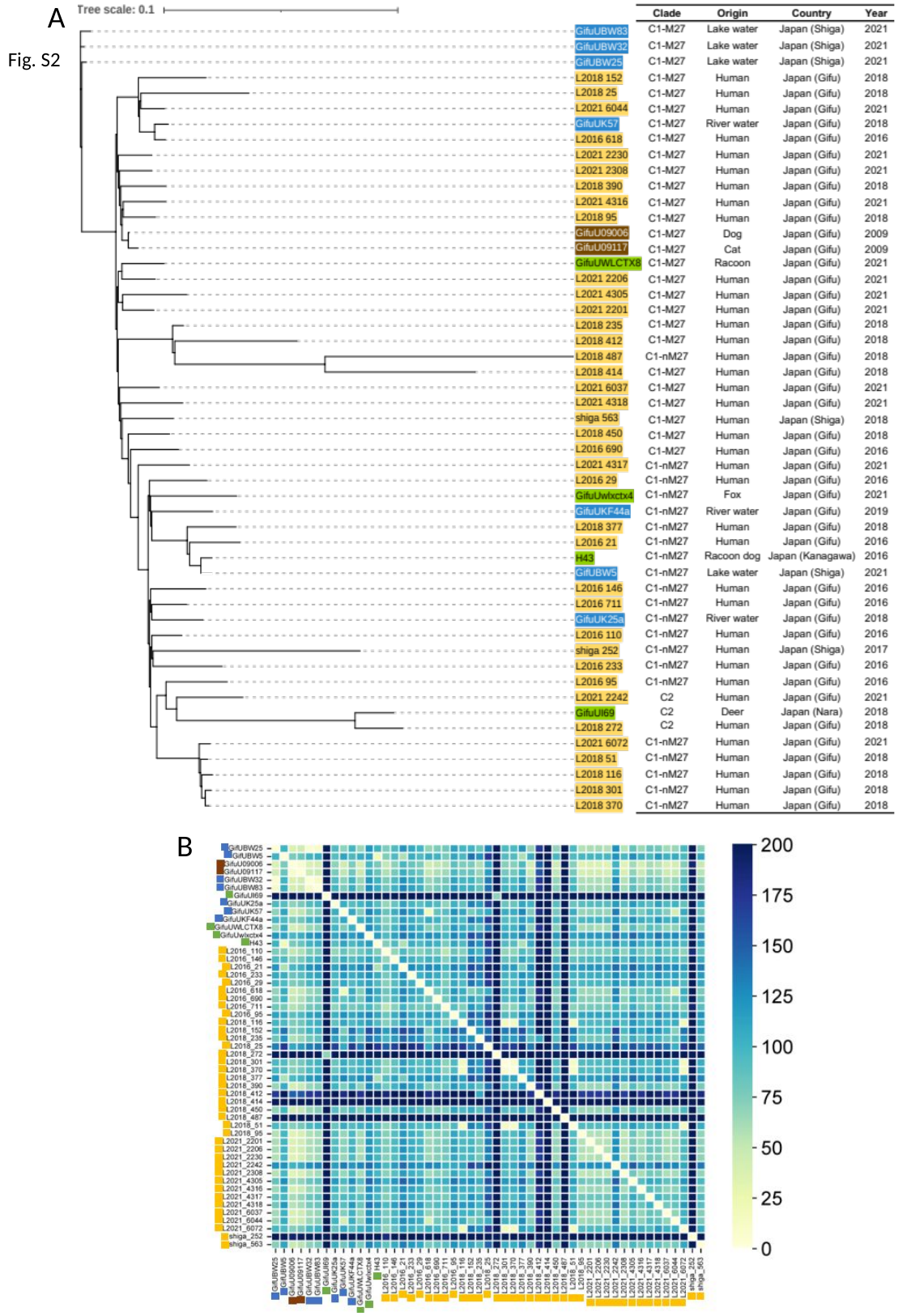

A
Fig. S2
B

Supplement: Supplementary material — Supplemental Fig. S1. Accessory genome analysis of ST131 isolated in Japan and other countries. Isolates highlighted in color are fluoroquinolone-resistant Escherichia coli isolates derived from specific regions (three neighboring prefectures, Gifu, Shiga, and Nara) of Japan. Isolates are shown in yellow (urine samples from human patients), brown (cat and dog feces), green (wildlife feces), and blue (river and lake water). The thresholds for the phylotypes and clusters were defined at tree-scale values of 0.01 and 0.005, respectively. ND, not determined; ST, sequence type. Parentheses in the ST and country columns indicate ST131 clades and prefectures, respectively. The phylogeny of the pan-genome analysis is not shown because it could not classify ST131 into several phylotypes and/or clusters owing to the high genomic similarity of identical clones. Supplemental Fig. S2. Core genome SNP analysis of ST131 isolated in Japan and other countries ST131 isolates belonging to phylotype I were subjected to accessory genome analysis (Supplemental Fig. S1) to determine the genetic relationship of ST131 isolates with a higher resolution using core genome SNP analysis. The results are shown as a phylogenetic tree based on core genome SNP (A) and matrix heatmap of the pairwise SNP distance (B). Isolates highlighted in color are fluoroquinolone-resistant Escherichia coli isolates derived from specific regions (three neighboring prefectures, Gifu, Shiga, and Nara) of Japan. Isolates are shown in yellow (urine samples from human patients), brown (cat and dog feces), green (wildlife feces), and blue (river and lake water). In the pairwise SNP distance, the matrix heatmap shows the number of SNP in the ST131 strains. [file mmc1.zip › mmc1.pptx]
